# Why base tautomerization does not cause errors in mRNA decoding on the ribosome

**DOI:** 10.1093/nar/gku1044

**Published:** 2014-10-28

**Authors:** Priyadarshi Satpati, Johan Åqvist

**Affiliations:** Department of Cell and Molecular Biology, Uppsala University, Biomedical Center, Box 596, SE-751 24 Uppsala, Sweden

## Abstract

The structure of the genetic code implies strict Watson–Crick base pairing in the first two codon positions, while the third position is known to be degenerate, thus allowing wobble base pairing. Recent crystal structures of near-cognate tRNAs accommodated into the ribosomal A-site, however, show canonical geometry even with first and second position mismatches. This immediately raises the question of whether these structures correspond to tautomerization of the base pairs. Further, if unusual tautomers are indeed trapped why do they not cause errors in decoding? Here, we use molecular dynamics free energy calculations of ribosomal complexes with cognate and near-cognate tRNAs to analyze the structures and energetics of G-U mismatches in the first two codon positions. We find that the enol tautomer of G is almost isoenergetic with the corresponding ketone in the first position, while it is actually more stable in the second position. Tautomerization of U, on the other hand is highly penalized. The presence of the unusual enol form of G thus explains the crystallographic observations. However, the calculations also show that this tautomer does not cause high codon reading error frequencies, as the resulting tRNA binding free energies are significantly higher than for the cognate complex.

## INTRODUCTION

The ribosome ensures high speed and accuracy in translation (1–4) by selecting the correct aminoacyl-tRNA (aa-tRNA) specific to the mRNA codon presented in the ribosomal A-site, from the pool of aa-tRNAs. Standard Watson–Crick base pairing (A with U and G with C) is at the heart of the selection process. It has been suggested that the ribosome recognizes correct codon-anticodon Watson–Crick geometry by its shape and achieves high accuracy by interaction with the so-called monitoring rRNA bases of the 30 S subunit, concomitant with a small 30S inter-domain movement (5). These monitoring bases, A1492, A1493 and G530, adopt significantly different conformations in the case of an empty A-site (6–8) and an A-site into which a tRNA (or anticodon stem-loop) has relaxed (5,9–11). In the former case the bases appear conformationally variable and are generally directed away from the decoding site, while upon tRNA binding they become ordered and interact with the minor groove of codon-anticodon minihelix. The monitoring bases have a tighter interaction with the first two codon-anticodon positions relative to the third (5,12), which partly explains the degeneracy of the genetic code. Hence, any deviation from standard Watson–Crick geometry at the first two codon positions resulting from near-cognate (A-C or G-U) mismatches will be strongly sensed by the monitoring bases, causing efficient rejection of the incorrect tRNA (2,4,13). Furthermore, tRNA modifications also have a prominent role in expanding the decoding capacity and maintaining fidelity (14–19) and is has, for example, been shown that modifications at residue 34 of the tRNA anticodon can both expand and restrict the ability to recognize multiple codons (14–18).

A key unresolved question in mRNA decoding on the ribosome regards the possibility of tautomerization of the codon-anticodon bases, which could potentially be a threat to high fidelity. Particularly for near-cognate A-C and G-U mismatches tautomerization of either of the bases could add an extra hydrogen bond to the pairing. In the former case (A-C) it is imino tautomers and in the latter (G-U) it is the enol forms that could cause decoding problems. Such tautomeric equilibria for free bases in solution are difficult to measure accurately due to the high prevalence of the standard amino and keto forms (20). However, quantum mechanical calculations (including solvation effects) clearly predict that it is easier to form the relevant C (imino) and G (enol) tautomers (6–8 kcal/mol) than those of A and U (>10 kcal/mol) (21–23). As far as A-C mismatches are concerned, it is also energetically more favorable to protonate the adenine at N1 (p*K*_a_ = 3.5) at neutral pH in solution (4.8 kcal/mol) than to invoke the imino form of cytosine. For the case of tRNA^Phe^_GAA_ misreading the serine UCU codon, with a second position A-C mismatch, recent molecular dynamics (MD) free energy calculations also showed that protonation of the anticodon A yielded almost isoenergetic tRNA binding compared to the unprotonated case (24). However, the predicted discrimination against the UCU codon with tRNA^Phe^_GAA_ accommodated in the A-site was about 7 kcal/mol in both cases, indicative of a very high second position fidelity. While there is no 3D structural information available for first and second position A-C codon-anticodon mismatches on the ribosome, a crystal structure has been determined with a tRNA^Trp^_CCA_ variant bound to UGA codon (25). This corresponds to a third position A-C mismatch and shows a single hydrogen bond between the bases. Both the structure and energetics of that complex was also well predicted by computer simulations (26,27).

Keto-enol tautomerization of a G-U base pair at the wobble position has, in fact, been suggested as a possible reason for the expanded genetic code readability of tRNA^Val^ carrying a 5-oxyacetic acid modified U at position 34 (18). This modification enables reading of all four valine codons and the crystal structure of the anticodon stem-loop complex with ribosome seems to indicate a Watson–Crick geometry of the G-cmo^5^U wobble base pair (18). However, if the same (G or U) enol tautomer would be energetically accessible at the first two codon positions this would obviously cause protein synthesis errors. Interestingly, recent crystal structures (11) of near-cognate aa-tRNAs bound to the 70S bacterial ribosome, and fully accommodated into the A-site, indicate that the first two base pairs are forced to adopt standard Watson–Crick geometry even with G-U mismatches. Although the resolution is moderate (3.1–3.4 Å) the electron density does not seem to allow any other reasonable interpretation. This means that either the crystal structures have captured a state with high repulsion between the standard G and U tautomers or that an enol state is actually being observed. At any rate, these near-cognate complexes must correspond to a state that is high in energy in order for the reading error to be low.

Here we address the codon reading energetics on the ribosome for G-U mismatches in the first two codon positions by extensive molecular dynamics free energy simulations (28). We examine misreading of the phenylalanine UUU codon by tRNA^Leu^ and tRNA^Ser^, both corresponding to G-U mismatches, in the first and second position, respectively. Besides the standard keto forms of the base pairs, the enols of both the codon and anticodon bases are considered, and their energetics evaluated using a combination of MD simulations and quantum chemical calculations. The results clearly show that enolization does not introduce unacceptably high error frequencies in codon reading. However, we find that the enol tautomer of the anticodon G can be equally or even more stable than the standard keto form for a G-U mismatch, although such a mismatch is still significantly higher in energy than a cognate A-U base pair.

## MATERIALS AND METHODS

### Molecular dynamics simulations

Initial coordinates for MD simulations of the fully accommodated (A/A) state were retrieved from the crystal structure with cognate tRNA^Phe^_GAA_ bound in the ribosomal A-site with the UUU codon (PDB ID: 3I8H) (10). The corresponding complexes with tRNA^Ser^_GGA_ and tRNA^Leu^_GAG_ were modeled by mutating A to G in the anticodon of the tRNA^Phe^ complex at position 35 and 36, respectively. Calculations were carried out both with (Supplementary Table S1) and without the 2-methylthio-N6-isopentenyladenosine (ms^2^i^6^A) modification at position 37 found in tRNA^Phe^. All MD simulations were performed with spherical simulation systems as implemented in the program Q (29). The simulation procedures were identical to those reported in earlier work (24,30). Spheres of radius 25 Å centered on the N1 atom of the first codon position were cut out from the crystal structures and solvated by a 37 Å radius water droplet, where water molecules at the sphere boundary were subjected to radial and polarization restraints according to the SCAAS model (29,31). Solute atoms 22–25 Å from the center were tightly restrained throughout the simulations leaving the inner 22Å radius sphere, which includes all A- and P-site atoms, fully flexible. Mg^2+^ ions were taken as described earlier (24,30) from the crystal structure and added to the system in order to obtain an overall neutral simulation sphere. Phosphate groups beyond 22Å from the simulation center were neutralized by scaling down the partial charges. The MD simulations were performed using CHARMM22 force field (32,33) with a 1 fs time step and a direct cut-off of 10 Å for non-bonded interactions, with electrostatic interactions beyond this cut-off treated by the local reaction field multipole expansion method (34). No cut-off was applied to the base that was mutated. Charges for the enol forms of G and U were derived so as to be compatible with the force field, utilizing minimally modified heteroatom charges from A and C as well as hydroxyl group parameters from tyrosine (Supplementary Figure S1).

**Figure 1.**
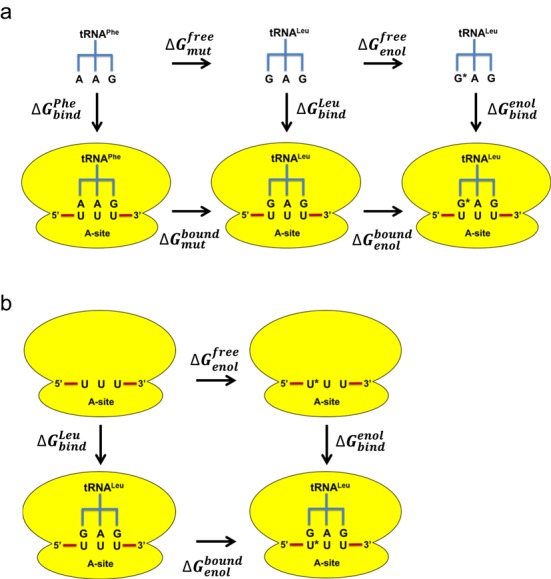
Thermodynamic cycles for evaluation of tRNA binding to the ribosome. (**a**) The cycle used to calculate relative binding free energies between different tRNAs to the same codon (UUU), where G* denotes the enol form of G. (**b**) Thermodynamic cycle used for evaluating the effect of tautomerization of the mRNA codon for a given tRNA, where U* denotes the enol form of U. Molecular dynamics free energy calculations are carried out along the horizontal legs in all cases and the final ΔΔ*G*_bind_ (Table 1, Figure 2) is obtained either from ΔΔ*G*_mut_ or ΔΔ*G*_enol_ (i.e. the differences between the bound and free legs) together with the Δ*G*_wat_ penalty for the enol forms (Table 1).

For the simulations of the tRNAs in water (Figure 1a), the tRNA molecule along with counter ions close to its backbone were taken from the model of the ribosome complex. The overall charge of the simulation sphere is neutral with 20 tRNA nucleotides and 10 Mg^2+^ ions included. Most of the counter ions are tightly bound to the tRNA backbone and none of them are close to the anticodon bases. The restraint region for the tRNA simulations was same as in the ribosome complex and the anticodon is fully water exposed and at the center of the simulation sphere. Because the anticodon bases that are mutated are fully exposed to water, the free energy results in these calculations are found to be very stable. Independent simulations starting with different initial velocities (see below) give <0.5 kcal/mol differences in mutation free energies despite the fact that the final counter ion positions, of course, can differ between different simulations.

It should also be emphasized here that there are distinct advantages with using the spherical simulation models (19,24,26) for the present type of free energy calculations. That is, with such a reduced simulation system it is possible to do many independent simulations that allow sufficiently good statistics to be attained. This is not only due to a smaller system size but also to the fact that large scale motions that occur on much longer time scales are suppressed. Such motions themselves are, of course, better studied with periodic boundary models including the entire ribosome, but will then require very long simulations for convergence. There is, however, considerable progress being made in the general area of nucleic acid MD simulations as described in two excellent recent reviews (35,36).

### Free energy calculations

Relative binding free energies were calculated with the free energy perturbation (FEP) method (28) as described earlier (26,30). Mutations of A to G and A to G^enol^ were performed for positions 35 and 36 of the tRNA anticodon, which make base pairs with first two codon positions of the mRNA. These simulations were done both for the tRNA bound to the ribosome programed with the UUU codon and for the free tRNA in water. This allows the relative binding free energies ΔΔ*G*_bind_ to be calculated via a standard thermodynamic cycle (Figure 1a). Each separate free energy calculation with the free and ribosome bound tRNAs involved with 51 discrete FEP windows and was repeated 8–10 times with different initial velocities from a Maxwell distribution, yielding a total of 10–30 ns of data collection for each such calculation. In order to explore the effects of enolization at the first and second codon position uracils, the same type of free energy calculations were also carried out for the mRNA programed ribosome with and without tRNA^Leu^ and tRNA^Ser^ bound to it, where the UUU codon was then mutated into U^enol^UU and UU^enol^U. The corresponding thermodynamic cycle for obtaining the relative binding free energies of the tRNAs to the keto and enol forms of the codon is shown in Figure 1b (see Ref. 37 for a similar thermodynamic cycle). In order to minimize the convergence errors of the FEP simulations we also explored which paths in Figure 1 yielded the most precise free energy estimates. Thus, for enolization of the anticodon base it was found that direct calculations of the A → G and A → G^enol^ paths were most efficient, and the G → G^enol^ results were thus obtained indirectly from the two former paths (Table 1). For the mRNA U → U^enol^ calculations we further used the double mutation path A-U → G-U^enol^ in the second codon position to improve convergence and, in that case, the G-U → G-U^enol^ result is indirectly calculated (Table 1).

**Table 1.**
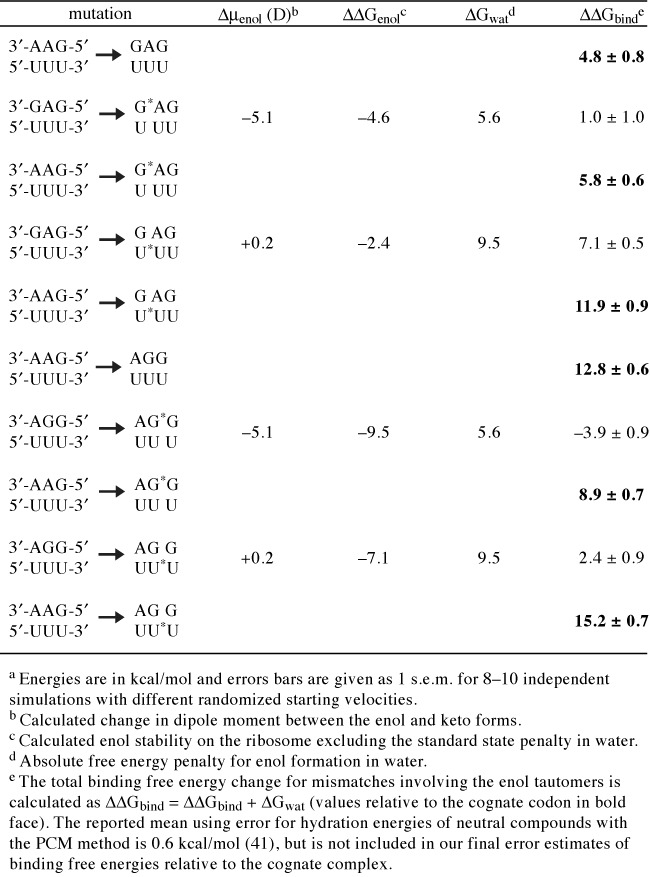
Energetic effects of base tautomerization on tRNA binding free energies^a^

Here, it is important to note while the thermodynamic cycles in Figure 1 directly give the relative tRNA binding strengths between the keto and enol forms of codon and anticodon, they do not include the absolute free energy cost of forming the enol in a solvated (water exposed) environment. This free energy term, which represents the cost of bringing the enol to the same standard state as the ketone, thus needs to be added as a correction to the calculated relative binding free energies. Note also that the mRNA codon bases are solvent exposed in absence of the A-site tRNA molecule, wherefore the solution energetics of enolization of the codon uracils should be a good approximation to the situation on the ribosome with vacant A-site (Figure 1b). Thus, to compute the corresponding free energy difference between the keto and enol forms of G and U in water, density functional calculations were carried out with the Gaussian09 software (38) using the M06–2X functional (39) (optimization with the 6−311++G** basis set) and the polarizable continuum model (PCM) (40,41) continuum solvent model. The N9 and N1 ring nitrogens of G and U, respectively, were capped by −CH_3_ groups in these calculations. An alternative approach here would be to evaluate only the gas-phase component by quantum mechanical calculations and the solvation contribution from explicit free energy calculations as done in Ref. 22, but the results therein were very similar with the PCM and FEP methods for the solvation term. The reported mean unsigned error of 0.6 kcal/mol for hydration free energies of neutral compounds with the PCM method (41) is also quite satisfactory for the present purposes.

## RESULTS

### Energetics of mismatches with standard tautomers

To explore the possible existence of rare G-U tautomers at the first two positions of the codon-anticodon pair we carried out extensive molecular dynamics free energy simulations of cognate and near-cognate ribosome complexes in both the keto and enol forms. The MD simulations were based on the crystal structure (10) with cognate tRNA^Phe^_GAA_ bound to the A-site of the 70S ribosome programed with a UUU codon. This structure has the tRNA fully accommodated in the so-called A/A conformation. It is further considered to give an accurate view of the proofreading stage of protein synthesis as the 30S monitoring bases A1492, A1493 and G530 are in tight interaction with the codon-anticodon minor groove (10). In order to compute relative binding free energies between different tRNAs competing for same UUU codon in the ribosomal A-site, we first used the FEP approach to mutate the cognate tRNA anticodon into near-cognate ones. Thus, the GAA anticodon (tRNA^Phe^) was mutated into GAG (corresponding to tRNA^Leu^) and GGA (corresponding to tRNA^Ser^) which yields G-U mismatches in the first and second codon position, respectively. Of the three tRNAs it is only tRNA^Phe^ that carries the ms^2^i^6^A modification at position 37 and we therefore report the results without this modification present. We have earlier shown that the main effect of ms^2^i^6^A37 in tRNA^Phe^ is to boost discrimination against a near-cognate first position mismatched codon in the initial selection of the EF-Tu ternary complex (A/T state), which is not relevant for the present situation (A/A state). However, as an additional check, we also carried out calculations with the modification present and the energetic results (Supplementary Table S1) are, in fact, very similar to those reported below.

**Figure 2.**
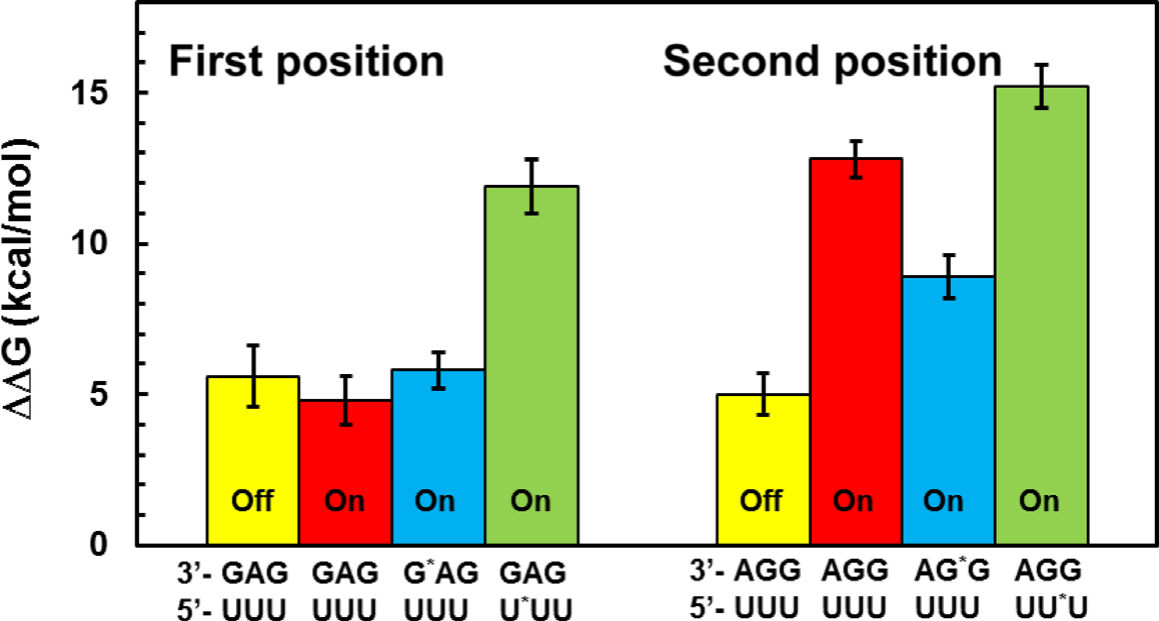
Calculated energetics of mismatches in the first and second codon positions. Calculated binding free energies of tRNA^Leu^_GAG_ and tRNA^Ser^_GGA_ (in kcal/mol) are given relative to the cognate tRNA^Phe^_GAA_ complex with the UUU codon. The enol forms of G and U are denoted G* and U*, respectively. Yellow bars denote calculations with the monitoring rRNA bases in their Off-state. Error bars, 1 s.e.m.

The energetic penalties of binding the standard keto forms of tRNA^Leu^ and tRNA^Ser^ to the UUU codon are predicted by the MD/FEP simulations to be 4.8 and 12.8 kcal/mol, respectively (Figure 2, Table 1). The value of about 5 kcal/mol discrimination against a first position G-U mismatch (tRNA^Leu^) appears reasonable in that it is somewhat smaller than found earlier for a more severe first position A-C mismatch (24). It corresponds to a reduced binding affinity compared to the cognate tRNA^Phe^ by factor of about 3000. However, the very strong penalty against tRNA^Ser^ appears unphysically large and would also seem incompatible with the observed binding of a second position G-U mismatched tRNA in crystal structures (11). That this strong discrimination has a significant contribution from the interaction with the monitoring bases in the minor groove (their ‘On-state’) was verified by repeating the calculations with these bases turned away to their Off-state (24). As expected, this yielded a much reduced ΔΔ*G*_bind_ = 5.0 kcal/mol for the second position mismatch with the UUU codon, i.e. for tRNA^Phe^ → tRNA^Ser^, but the fact that the monitoring bases are crystallographically observed in the On-state for the near-cognate complexes still remains unexplained. It may also be noted that a similar value of ΔΔ*G*_bind_ = 5.6 kcal/mol is predicted for tRNA^Phe^ → tRNA^Leu^ in the Off-state (Figure 2), in agreement with earlier work (24).

### Energetics of mismatches with enol tautomers

The results from the free energy calculations on the enol forms of mismatched first and second position G-U pairs are also summarized in Figure 2. There it can be seen that enolization of U in the mRNA yields less favorable binding free energies of the mismatched tRNA^Leu^ and tRNA^Ser^ than the keto forms, resulting in significantly stronger discrimination against the near-cognate complexes. Enolization of G in the tRNA, on the other hand, does not cause large additional penalties and, in the case of tRNA^Ser^ with a second position mismatch, it actually decreases the discrimination by about 4 kcal/mol. For the first position mismatch with tRNA^Leu^ the corresponding effect is a moderate selectivity increase of 1 kcal/mol. Hence, it is clear that for the mismatched complex of tRNA^Ser^ with the UUU codon, enolization of the second position (anticodon) G yields the most favorable energetics among the possible tautomers. However, it is important to point out here that ‘enolization does not alleviate the high selectivity against incorrect tRNAs’, either of the first two positions, since the discrimination is still very high. Further, that the second position should be mostly affected by taumerization is logical in view of the fact that it sequestered between the two other base pairs and therefore has more limited possibilities of structural relaxation. This is also in line with earlier computational and experimental observations of an intrinsically higher selectivity in the second position (4,24,42), which also may be reflected by the genetic code structure where the second position is the main determinant of physico-chemical properties of the coded amino acid.

The net effects of tautomerization on tRNA selectivity (Figure 2) are the result of compensating factors for which the detailed energetics is given in Table 1. In all cases, i.e. both G and U enolization and in the first and second position, does the ribosome actually stabilize the enol tautomer of the mRNA or tRNA (ΔΔ*G*_enol_ in Table 1). However, this stabilization is counterbalanced by the high energetic cost of forming the enol tautomer in water (Δ*G*_wat_ in Table 1), which is equivalent to bringing this form to a standard state of 1 M (the same as the keto form). In the case of U tautomerization the latter penalty is predicted to be about 4 kcal/mol higher than that of G and this is the basic reason for why the enol form U is unlikely to ever play any significant role in G-U base pairing. The present calculations of Δ*G*_wat_ are also in reasonable agreement with earlier quantum mechanical results for the energetics of enolization in continuum solvent models of water (21,22).

### Structural effects of base tautomerization

**Figure 3.**
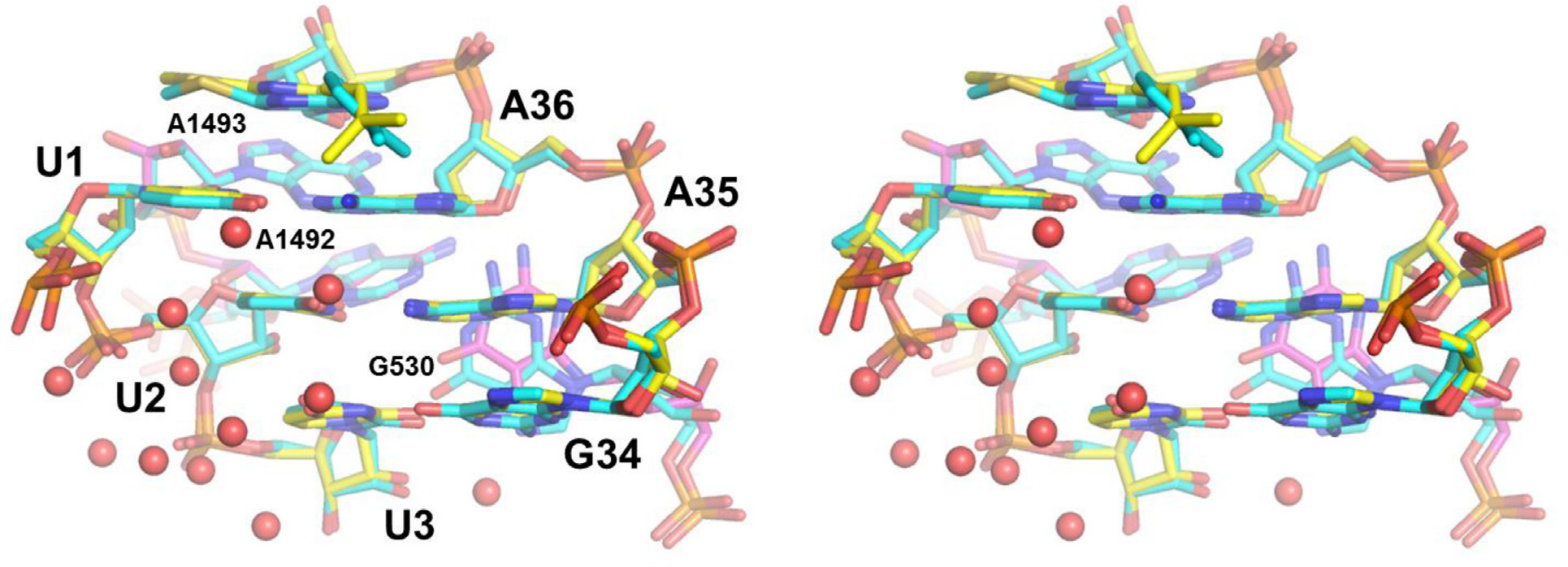
Comparison of the average MD and crystal structure for the cognate complex. Stereo view of the average MD structure (yellow carbons with the monitoring bases depicted with magenta carbons) overlayed on the crystal structure (10) of the cognate tRNA^Phe^_GAA_ complex (cyan carbons). Water molecules in the vicinity of the codon-anticodon pair are shown as red spheres.

**Figure 4.**
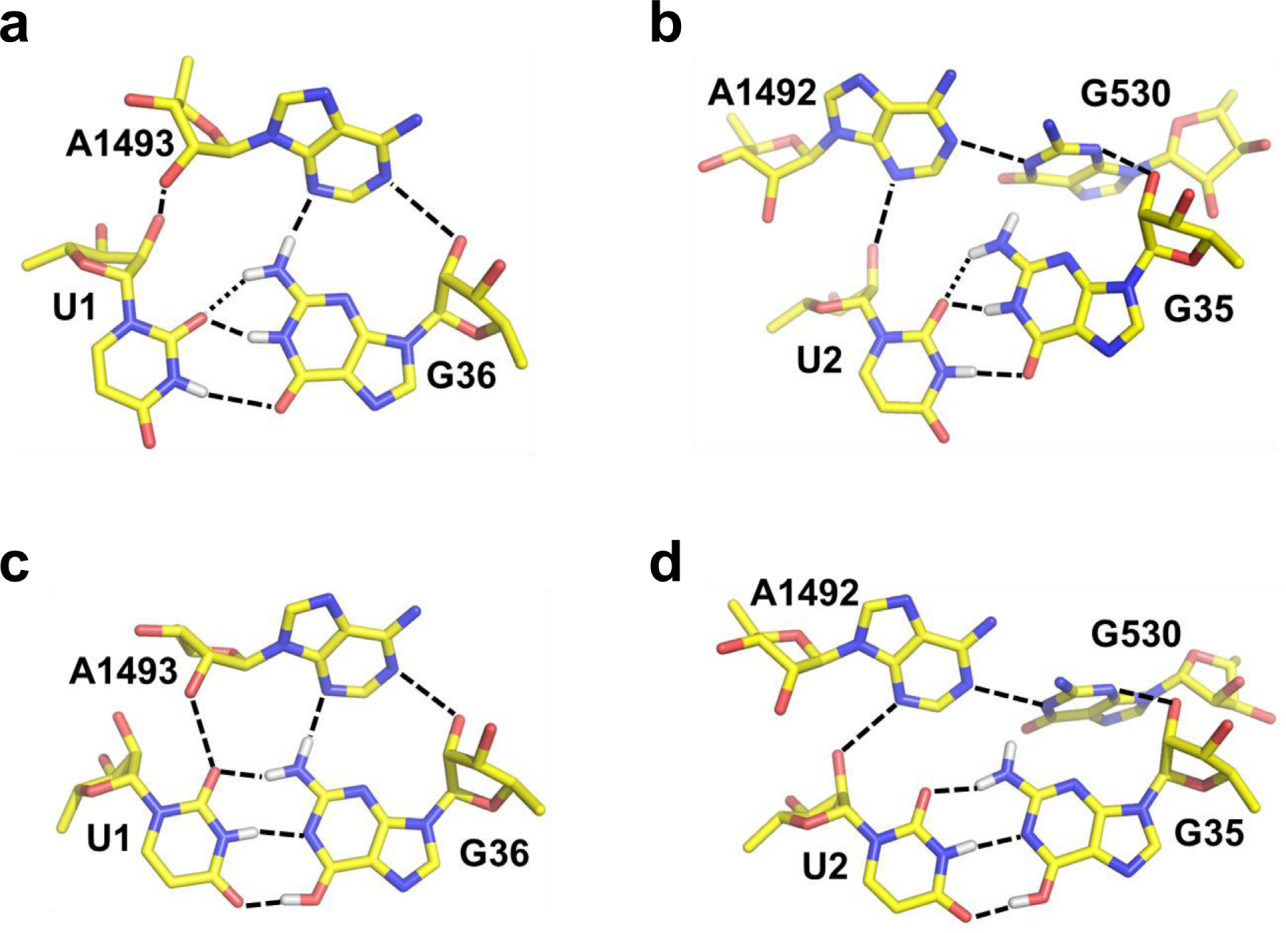
Average MD structures of first and second position G-U mismatches. (**a**) First position G-U mismatch with the standard keto form of the anticodon for tRNA^Leu^_GAG_ binding to the UUU codon. (**b**) Second position G-U mismatch with tRNA^Ser^_GGA_ and the standard keto form of the anticodon. (**c**) First position mismatch with tRNA^Leu^_GAG_ and the enol form of G36 in the anticodon. (**d**) Second position mismatch with tRNA^Ser^_GGA_ and the enol form of G35 in the anticodon. The key hydrogen bonds are drawn with dashed lines and the weaker (somewhat longer distances) hydrogen bonds between the anticodon amine and O6 of the codon U are shown as dotted lines in panels (**a**,**b**).

The MD simulations of the cognate crystal structure yield an excellent agreement with the observed conformation of the decoding region, with an r.m.s. heavy atom coordinate deviation for the fully mobile inner 22 Å sphere of 1.0 Å with respect to the 3I8H structure (10). The average MD structure (Figure 3) shows that the minor groove of the codon-anticodon minihelix is completely dehydrated due to solvent exclusion caused by the On-conformation of the monitoring bases (the crystal structures do not have any water molecules refined). This phenomenon was been also observed in earlier MD simulations (24) and is the main reason for why the monitoring bases can increase the penalty of mismatches in their On-conformation, since no water molecules can enter to compensate for missing hydrogen bond interactions in non-cognate complexes (24). However, it is interesting to note that, as opposed to the case of a first position A-C mismatch (24), the effect of switching On/Off the monitoring bases is negligible for a G-U mismatch as in the case of tRNA^Leu^ (Figure 2). This phenomenon is explained by the hydrogen bond that can be formed between the amino group of G36 in the tRNA and the A1493 base (Figure 4a), which is also observed experimentally for cognate first position G-C pairs (11). That is, in the On-conformation of A1493 this hydrogen bond can actually compensate for the suboptimal base pairing.

The G-U mismatches naturally lead to distortions of the standard (A-U) geometry and corresponding average MD structures, in the standard keto form, are shown in Figure 4. Besides the most prevalent G-U interaction with two hydrogen bonds (Figure 4a and b), another type of conformation is also occasionally seen in the MD simulations where a water molecule can bridge the hydrogen bond between N3 of U and O6 of G. Furthermore, it is apparent that the change in shape of the minor groove due to the G-U pair is sensed differently by the ribosome in the first and second positions. As noted above, in the former case the amine group of the anticodon G can form a hydrogen bond with N3 of A1493 (Figure 4a), whereas no such hydrogen bonding interaction for the amine is possible in the second position. Hence, it is logical that the energetic penalty is larger for a mismatched second position anticodon G, with its amine group is in the minor groove of the codon-anticodon minihelix. This is because A1492 and G530 efficiently exclude any solvent in their On-conformation, thereby causing partial desolvation of the base.

The simulations of the G^enol^-U and G-U^enol^ tautomers confirm a standard Watson–Crick geometry of these ribosomal complexes, with three distinct hydrogen bonds between the bases. The average MD structures of the two alternative enol forms (G^enol^-U and G-U^enol^), in both codon positions, are virtually identical apart from the enolic proton position and are shown in Figure 4 for the G^enol^-U cases. The fact that the O…H−O hydrogen bonds are generally stronger than those involving nitrogen is also reflected by it being slightly shorter (2.6–2.7 Å O…O distance), in agreement with a recent higher resolution crystal structure of a DNA polymerase complex with a G-T mismatch showing Watson–Crick geometry (43). In addition to the base pair interactions, the crystallographically observed (10) hydrogen bonds to the O2′ and N3 of A1493 can be maintained by the first position G^enol^-U pair. In the second position, no such hydrogen bonding to the ribosome is possible (Figure 4d), which makes the three base pair hydrogen bonds energetically more important. This appears to the main reason for why the net stabilization of the enol form is larger in the second position.

Since the structures of the G^enol^-U and G-U^enol^ complexes are identical to each other in both codon positions (only differing in the enolic proton position), the energetics of their interactions with the ribosome are very similar. However, since the enolization of U increases the dipole moment of the base, while enolization of G decreases it (see Table 1 and (21)), the solvation energies of the bases in the reference states (Figure 1) go in opposite directions. This is another factor that contributes to the less favorable energetics of U enolization and is reflected by the less negative values of ΔΔ*G*_enol_ in Table 1 for both the first and second position G-U^enol^ compared to their respective G^enol^-U form. That is, besides the overall energy of forming U^enol^ in water being higher than that for G^enol^ (Δ*G*_wat_ in Table 1, which also includes the electronic contribution), the solvation energy is more negative relative to the keto form than for G^enol^ (for which it is positive). These results are also in agreement with earlier calculations (21,22).

## DISCUSSION

The idea that tautomerization of nucleic acid bases can be a source of error in the otherwise specific base pairing mechanism dates back to Watson–Crick, who proposed this as an origin of spontaneous mutation in DNA replication (44). With regard to mRNA translation the problem is the same, namely, that less prevalent tautomeric forms could potentially change the specific codon-anticodon pairing and cause errors in protein synthesis. The unique crystal structures obtained by Yusupova *et* *al*. (11) of non-cognate aa-tRNAs bound to the ribosome, with mismatches in the first and second codon positions, show base pair geometries indicative of tautomerization. We also verified this by calculating electron density maps from the deposited structure factors. It should further be noted here that the possibility of ionization of either G (N1) or U (N3), with normal p*K*_a_'s around 9.4, cannot explain the crystal structures with close oxygen-oxygen contacts between the bases. A similarly mismatched G-U structure was also observed in a recent crystal structure of an error-prone variant of DNA polymerase λ (43). Since this type of structures are apparently possible to trap crystallographically, this immediately raises the question of whether their energetics is sufficiently prohibitive in order to avoid errors.

Our computer simulations unambiguously show that enolization of G-U base pairs in the first two codon positions on the ribosome does not cause any significant error in the specificity of tRNA binding. That is, the ribosome complexes with fully accommodated aa-tRNAs, corresponding to the proofreading stage of protein synthesis (11), are predicted to disfavor the enol forms of non-cognate tRNAs (tRNA^Leu^ and tRNA^Ser^) by at least a factor of about 10 000 compared to the cognate case (tRNA^Phe^). So it is clear that tautomerization does not alleviate the high discrimination against incorrect substrates for peptide elongation. Our results also show that, as far as G-U mismatches are concerned, the enol tautomer of guanine is far more stable than that of uracil, both in aqueous solution and on the ribosome. The enol form of G is, in fact, predicted to be more stable than the keto form for a second position G-U mismatch, while it is approximately isoenergetic with the ketone in the first position of the codon-anticodon pair. In both cases, however, is the cognate A-U pair highly favored.

The present calculations are thus in agreement with the observed crystal structures of G-U mismatches on the ribosome (11) in the sense that, given that such complexes can be trapped, the enolic forms are indeed predicted to be energetically accessible (first position) or even the most stable (second position). The question of how it can be possible to trap such complexes in the first place is perhaps even more interesting. That is, how can you trap a structure that is 6–9 kcal/mol higher in free energy than the cognate complex? Here, it should be noted that besides a high excess of the non-cognate tRNAs over ribosomes, the crystals were also obtained with a high Mg^2+^ concentration (11). This is known to increase the error frequency in protein synthesis and the rate of both GTP hydrolysis and peptide bond formation with non-cognate tRNAs (4). Further, the mechanism whereby increased Mg^2+^ concentration speeds up both cognate and non-cognate reactions at the expense of losing accuracy seems to be that the On-state of the monitoring rRNA bases is stabilized irrespective of the A-site substrate (4,11,45). For example, the crystal structures (11) with G-U mismatches (PDB IDs: 3UYD and 3UZG) show a Mg^2+^ ion with strong electron density in helix 44, in the region occupied by A1492 and A1493 in their Off-state on ribosomes with empty A-sites (6–8). This was also verified from our calculated electron density maps based on the deposited structure factors (11). It thus appears that the monitoring bases can be titrated from the Off- to the On-state for non-cognate substrates by increased Mg^2+^ concentration. This is similar to the error-inducing mechanism of paramomycin which also binds in this region of helix 44 and drives the monitoring bases to their On-state (5,45,46).

Hence, in combination with the low temperature cryo-conditions used in crystallographic data collection it is perhaps not so surprising that this type of complexes can be trapped. It may further be noticed that under similarly high Mg^2+^ concentration conditions the dissociation of cognate aa-tRNAs from the ribosomal A-site has been measured as exceedingly slow (47). These off-rates are on the order of 10^−4^ s^−1^ at 20°C, which implies an exit free energy barrier of about 23 kcal/mol. Hence, even if the corresponding non-cognate exit barriers are 6–9 kcal/mol lower than this value the corresponding off-rates would indeed be small at the crystallographic cryo-temperature (100 K).

The calculations reported herein also confirm our earlier conclusions regarding the key role played by the monitoring bases, A1492, A1493 and G530, in maintaining a high fidelity (24). Thus their principal effect, in their On-state, is to exclude solvent from the minor groove side of the codon-anticodon minihelix. This causes the penalty for unsatisfied hydrogen bonds to become significantly larger than when the bases are in their Off-state and water molecules can compensate for such missing hydrogen bonds. However, the case with a G in position 36 of the tRNA anticodon appears somewhat special since this base, both in cognate G-C and near-cognate G-U complexes (11), can form a seemingly critical hydrogen bond to A1493 (Figure 4). This interaction is found here to make the On-state much less discriminatory for such a G-U pair than for a corresponding first position A-C mismatch (24). Conversely, for a G-U mismatch in the second position we find that the discrimination is much stronger in the On-state and similar in magnitude to that of A-C mismatches (24), as no compensating hydrogen bonds can be formed. This situation would thus lead to the prediction that discrimination against misreading a first position U would be similar also for the tRNAs coding for Pro, His, Gln and Arg that carry a G in position 36, at least as far as the proofreading stage is concerned. With regard to second position misreading of the UUU codon by tRNA^Ser^, it is also possible that the actual pathway when such a rare event occurs never involves the On-state of the monitoring bases observed crystallographically, but instead progresses through the Off-state with a lower energetic penalty.

As argued earlier (24), the solvent exclusion effect of the monitoring rRNA bases leads to the existence of both low selectivity Off-states and high selectivity On-states, where the latter seem to mainly pertain to the second codon position. These high selectivity states will in practice very rarely be populated by non-cognate tRNAs under physiological conditions, simply because they are too high in energy and, consequently, rejection from the ribosome instead becomes the preferred route for incorrect tRNAs. These high-energy states are thermodynamically hidden in the sense that they are difficult to probe experimentally but they are an essential component of the machinery that ensures high accuracy in protein synthesis.

## SUPPLEMENTARY DATA

Supplementary Data are available at NAR Online.

SUPPLEMENTARY DATA
