# Supplementary material for: Why base tautomerization does not cause errors in mRNA decoding on the ribosome
Source: Nucleic Acids Res. 2014 Oct 28;42(20):12876–84. doi: 10.1093/nar/gku1044 (PMC4227757; doi:10.1093/nar/gku1044)
Supplement: SUPPLEMENTARY DATA [file supp_42_20_12876__index.html]

Why base tautomerization does not cause errors in mRNA decoding on the ribosome — Why base tautomerization does not cause errors in mRNA decoding on the ribosome — SUPPLEMENTARY DATA 

# Why base tautomerization does not cause errors in mRNA decoding on the ribosome

## SUPPLEMENTARY DATA

**Files in this Data Supplement:**

- SUPPLEMENTARY DATA
