# Supplementary material for: Why base tautomerization does not cause errors in mRNA decoding on the ribosome
Source: Nucleic Acids Res. 2014 Oct 28;42(20):12876–84. doi: 10.1093/nar/gku1044 (PMC4227757; doi:10.1093/nar/gku1044)
Supplement: SUPPLEMENTARY DATA [file supp_gku1044_nar-02387-f-2014-File003.pdf]

Supplementary Figure and Table

Why base tautomerization does not cause errors in mRNA decoding on the ribosome

Priyadarshi Satpati and Johan Åqvist

Department of Cell and Molecular Biology, Uppsala University,
Biomedical Center, Box 596, SE-751 24 Uppsala, Sweden

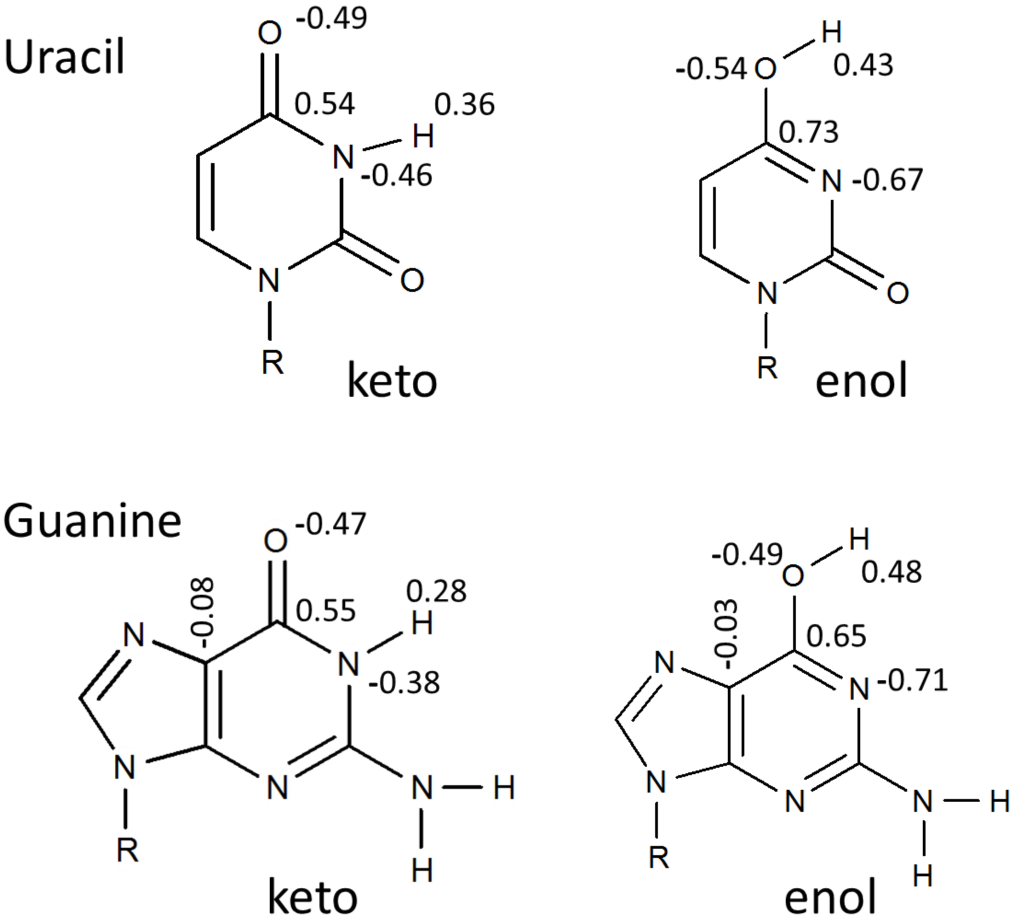

Supplementary Figure S1. Force field charges for keto and enol forms of U and G.

Standard CHARMM22 partial charges (32,33) are shown for the keto forms (left) together with the modified charges for the enol tautomers (right). The latter were adapted from the corresponding standard charges of tyrosine (hydroxyl group), cytosine and adenine (deprotonated ring nitrogens).

Supplementary Table S1.

Energetic effects of base tautomerization including the ms^2i^6A37 tRNA modification. [a]

mutation	$\Delta\mu_{enol}$ (D)[b]	$\Delta\Delta G_{enol}$[c]	ΔG_{wat}[d]	$\Delta\Delta G_{bind}$[e]
3'-AAG-5' → GAG<br>5'-UUU-3' → UUU				**4.3 ± 0.7**
3'-GAG-5' → G*AG<br>5'-UUU-3' → U UU	–5.1	–5.4	5.6	0.2 ± 0.9
3'-AAG-5' → G*AG<br>5'-UUU-3' → U UU				**4.5 ± 0.6**
3'-GAG-5' → G AG<br>5'-UUU-3' → U*UU	+0.2	–3.5	9.5	6.0 ± 0.4
3'-AAG-5' → G AG<br>5'-UUU-3' → U*UU				**10.3 ± 0.8**
3'-AAG-5' → AGG<br>5'-UUU-3' → UUU				**14.1 ± 1.1**
3'-AGG-5' → AG*G<br>5'-UUU-3' → UU U	–5.1	–8.9	5.6	–3.3 ± 1.4
3'-AAG-5' → AG*G<br>5'-UUU-3' → UU U				**10.8 ± 0.9**
3'-AGG-5' → AG G<br>5'-UUU-3' → UU*U	+0.2	–8.7	9.5	0.8 ± 1.3
3'-AAG-5' → AG G<br>5'-UUU-3' → UU*U				**14.9 ± 0.7**

[a] Energies are in kcal/mol and errors bars are given as 1 s.e.m. for 8-10 independent simulations with different randomized starting velocities. [b] Calculated change in dipole moment between the enol and keto forms. [c] Calculated enol stability on the ribosome excluding the standard state penalty in water. [d] Absolute free energy penalty for enol formation in water. [e] The total binding free energy change for mismatches involving the enol tautomers is calculated as $\Delta\Delta G_{bind} = \Delta\Delta G_{enol} + \Delta G_{wat}$ (values relative to the cognate codon in bold face). The reported mean unsigned error for hydration energies of neutral compounds with the PCM method is 0.6 kcal/mol (41), but is not included in our final error estimates of binding free energies relative to the cognate complex.
